# Risk factors for coronary artery disease in patients undergoing elective coronary angiography in Jordan

**DOI:** 10.1186/s12872-017-0620-4

**Published:** 2017-07-11

**Authors:** Abdel-Ellah Al-Shudifat, Asgeir Johannessen, Mohammed Azab, Amjad Al-Shdaifat, Suhad Sameer AbuMweis, Lana M. Agraib, Reema F. Tayyem

**Affiliations:** 1 Faculty of Medicine, The Hashemite University, Zarqa, Jordan; 2 Centre for Imported and Tropical Diseases, Oslo University Hospital, Ullevål, PO Box 4956, 0424 Oslo, Norway; 3 Faculty of Allied Health Sciences, The Hashemite University, Zarqa, Jordan; 4 Faculty of Agriculture, The University of Jordan, Amman, Jordan

**Keywords:** Coronary heart disease, Risk factors, Smoking, Diabetes mellitus, Middle East

## Abstract

**Background:**

Unhealthy lifestyle factors such as smoking, obesity, inactivity and type 2 diabetes are endemic in the Middle East. The public health consequences might be detrimental; however, local studies on risk factors for coronary artery disease (CAD) are scarce.

**Methods:**

Patients referred for coronary angiography at a tertiary hospital in Amman, Jordan, between January and December 2015, were included in this study. Risk factors for CAD were assessed in a multivariate logistic regression model, and presented as odds ratio (OR) with 95% confidence interval (CI).

**Results:**

Among 557 participants, 356 (63.9%) had CAD and 201 (36.1%) had a normal cardiogram. The majority (*n* = 395, 70.9%) were male, and median age was 55 years (interquartile range 47–64). Two-hundred-and-fifteen (38.6%) individuals reported previous diabetes, and 287 (51.5%) were current or previous smokers. In multivariate analysis, male gender (OR 3.7, 95% CI 2.3–6.0), age (45–54 years: OR 4.8, 95% CI 2.7–8.5; 55–64 years: OR 6.0, 95% CI 3.2–11.4; ≥65 years: OR 15.7, 95% CI 7.8–31.3), previous diabetes (OR 2.6, 95% CI 1.7–4.1) and current/previous smoking (OR 2.1, 95% CI 1.3–3.4) were significant predictors of CAD.

**Conclusions:**

Age, gender, diabetes and smoking were strong and significant risk factors for CAD in Jordan. Public health interventions to reduce the prevalence of smoking and diabetes are urgently needed.

## Background

Cardiovascular disease (CVD) is the leading cause of death globally, and claimed an estimated 17.9 million lives in 2015 [1]. Age, gender, smoking, obesity, dyslipidemia, physical inactivity, hypertension and diabetes mellitus (DM) are established risk factors for CVD [2–5]. Most studies on predictors of CVD, however, have been carried out in Europe and North America, and little is known about the relative contribution of these risk factors in the Middle East.

Whilst the incidence of CVD is declining in many parts of the world [6], several lifestyle factors render the Middle East vulnerable to CVD in the coming decades. First, the region is home to one of the most obese populations in the world. In Jordan, it is estimated that 65.9% of adults above 18 years are either overweight or obese [7]. Second, as a consequence of the obesity epidemic, DM is steadily increasing. In 2014, an estimated 13.7% of the adult population in the region had DM, compared to 5.9% in 1980 [8]. Third, tobacco use is more popular in the Middle East than elsewhere in the world, and the World Health Organization (WHO) estimates that 41.0% of Jordanians above the age of 15 years are smokers [9]. Finally, a sedentary lifestyle is widespread in the region, illustrated by a recent study from 10 Middle Eastern countries, where only 19% of adolescents reported taking part in any sort of physical activity [10].

The aim of the present study was to assess risk factors for coronary artery disease (CAD) in a Middle Eastern population. Results from this study could eludicate the contribution of modifiable lifestyle factors to the burden of CAD, and hence pave the road for effective preventive measures relevant to the region.

## Methods

### Study setting and participants

Prince Hamzah Teaching Hospital is a tertiary referral hospital in Amman, Jordan. Participants for the present study were recruited from the catheterization section of the cardiology department between January and December 2015. The department offers elective coronary angiography services to patients with clinical suspicion of coronary artery disease (stable angina, ischemic heart disease, chest pain, positive cardiac stress test), whereas patients with acute coronary syndrome are treated elsewhere and therefore not included in the present study. Individuals who were pregnant or lactating, or who suffered from kidney disease, liver disease or gastrointestinal disease were excluded. Socio-demographic data, past medical history and smoking status were recorded by trained research assistants using standardized questionnaires. All patients provided written informed consent to participate in the study. The study was approved by the pertinent Institutional Review Board Ethics Committee.

### Coronary angiography

The procedure was performed by trained invasive cardiologists using standard technique. In brief, the catheter was inserted into the radial artery using a Seldinger technique, and the tip was advanced to the aortic sinus cusp. X-ray images of the transient radio-contrast distribution within the coronary arteries were carried out to visualize the arterial tree. The degree of obstruction was estimated as percentage of the arterial lumen by comparing the area of narrowing to an adjacent normal artery.

### Statistical analysis

The outcome of interest was CAD, defined as any obstruction (partial or complete) of the coronary arteries. Differences in baseline variables between patients with and without CAD were estimated using Chi-square tests. The Odds Ratios (OR) for CAD were estimated using binary logistic regression models. Variables with a *p*-value below 0.10 in univariate analysis were included in the multivariate model using a forward stepwise method. Multicollinearity was excluded using Spearman’s correlation coefficient with a cutoff at 0.7. Data were analysed using SPSS version 23.0 (SPSS Inc., Chicago, IL, USA). All tests were two-sided and the significance level was set at *p* < 0.05.

## Results

### Baseline characteristics

A total of 557 participants who underwent coronary angiography were included in the study. Of these, 356 (63.9%) had CAD and 201 (36.1%) had a normal cardiogram. Out of 356 patients with CAD, 116 (32.6%) underwent stent placement during the diagnostic procedure.

The majority of participants (*n* = 395, 70.9%) were male, and median age was 55 years (interquartile range 47–64). Previous DM, hypertension and dyslipidemia was reported by 215 (38.6%), 264 (47.4%) and 32 (5.7%) study participants, respectively. Two-hundred-and-eighty-seven individuals (51.5%) were current or previous smokers. Table 1 shows patient characteristics grouped by presence or absence of CAD.

**Table 1:** Baseline characteristics among individuals referred for coronary angiography, Amman, Jordan

	Total N (%)	CAD (*n* = 356) N (%)	Normal (*n* = 201) N (%)	*P* ^a^
Gender				
Male	395 (70.9)	282 (79.2)	113 (56.2)	<0.001
Female	162 (29.1)	74 (20.8)	88 (43.8)	
Age (years)				
< 45	96 (17.2)	31 (8.7)	65 (32.3)	<0.001
45–54	180 (32.3)	117 (32.9)	63 (31.3)	
55–64	148 (26.6)	101 (28.4)	47 (23.4)	
> =65	133 (23.9)	107 (30.1)	26 (12.9)	
Marital status				
Married	515 (92.6)	329 (92.7)	186 (92.5)	0.952
Single/Divorced/Widowed	41 (7.4)	26 (7.3)	15 (7.5)	
Education				
Illiterate	50 (9.0)	36 (10.2)	14 (7.0)	0.610
Primary	239 (43.1)	153 (43.2)	86 (42.8)	
Secondary	145 (26.1)	90 (25.4)	55 (27.4)	
Higher	121 (21.8)	75 (21.2)	46 (22.9)	
Employment				
Yes	236 (42.4)	144 (40.4)	92 (45.8)	0.222
No	321 (57.6)	212 (59.6)	109 (54.2)	
Family history of CVD				
Yes	199 (35.7)	124 (34.8)	75 (37.3)	0.557
No	358 (64.3)	232 (65.2)	126 (62.7)	
Smoking				
Current/previous	287 (51.5)	202 (56.7)	85 (42.3)	0.001
No	270 (48.5)	154 (43.3)	116 (57.7)	
Hookah smoking				
Current/previous	62 (11.1)	37 (10.4)	25 (12.4)	0.461
No	495 (88.9)	319 (89.6)	176 (87.6)	
Diabetes mellitus				
Yes	215 (38.6)	160 (44.9)	55 (27.4)	<0.001
No	342 (61.4)	196 (55.1)	146 (72.6)	
Hypertension				
Yes	264 (47.4)	176 (49.4)	88 (43.8)	0.199
No	293 (52.6)	180 (50.6)	113 (56.2)	
Hyperlipidemia				
Yes	32 (5.7)	20 (5.6)	12 (6.0)	0.864
No	525 (94.3)	336 (94.4)	189 (94.0)	

^a^Chi-square test

*CAD* coronary artery disease, *CVD* cardiovascular disease

### Risk factors for coronary artery disease

In univariate analysis, male gender, age 45 years or older, previous DM and current/previous smoking were significant predictors of CAD. Previous hypertension, previous dyslipidemia and a family history of CVD were not significantly associated with CAD.

Gender, age, DM and smoking all remained significant predictors of CAD in multivariate analysis. The strongest association was found for age; patients who were 65 years or older had more than 15 times the odds of CAD compared to those who were younger than 45 years (Table 2).

**Table 2:** Associations between baseline variables and coronary artery disease in adults undergoing coronary angiography, Amman, Jordan

	OR (95% CI)^a^	*P*	AOR (95% CI)^b^	*P*
Gender				
Female	1		1	
Male	3.0 (2.0–4.3)	<0.001	3.7 (2.3–6.0)	<0.001
Age (years)				
< 45	1		1	
45–54	3.9 (2.3–6.6)	<0.001	4.8 (2.7–8.5)	<0.001
55–64	4.5 (2.6–7.8)	<0.001	6.0 (3.2–11.4)	<0.001
> =65	8.6 (4.7–15.8)	<0.001	15.7 (7.8–31.3)	<0.001
Smoking				
No	1		1	
Current/previous	1.8 (1.3–2.5)	0.001	2.1 (1.3–3.4)	0.001
Diabetes mellitus				
No	1		1	
Yes	2.2 (1.5–3.1)	<0.001	2.6 (1.7–4.1)	<0.001

^a^Univariate logistic regression model

^b^Multivariate logistic regression model, adjusted for the other variables listed

*OR* odds ratio, *AOR* adjusted odds ratio, *CI* confidence interval

**Fig. 1:**
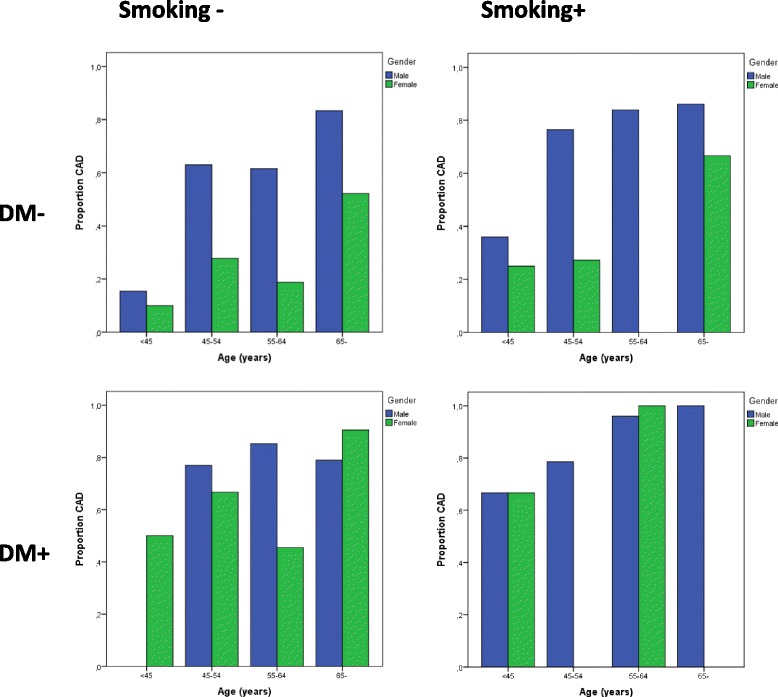
Proportion of patients with coronary artery disease among 557 individuals referred for coronary angiography

Figure 1 shows the proportion of participants with CAD, grouped by gender, age, DM and smoking. The probability of CAD ranged from 10.0% in non-diabetic, non-smoking women below 45 years, to 100.0% in diabetic male smokers above 65 years. Notably, among smokers with DM, both men and women, the probability of CAD was high in all age groups, even among those below 45 years, underscoring the importance of lifestyle factors in this population.

## Discussion

Male gender, increasing age, DM and smoking were strong and significant predictors of CAD in this study. This is one of few studies from the region to assess risk factors for CAD in people undergoing coronary angiography, and hence, provides new insight into the relevance of traditional risk factors in a Middle Eastern setting.

Previously, only a few similar studies from countries in the region have been published. Al-Kateb and colleagues reported that body mass index, age and smoking were significantly associated with CAD in a cohort of 192 male patients referred for catheterization in (pre-war) Syria [11]. In a more recent study from Iraq, Mohammad and colleagues found that male gender, smoking, hypertension, hyperlipidemia and a family history of CAD were associated with premature CAD, i.e. in patients younger than 45 years for men and 55 years for women [12]. Taken together, our results and previous studies suggest that traditional risk factors for CAD are of importance also in a Middle Eastern setting.

In our study, most of the patients with CAD had one or more risk factors. Current or previous smoking was reported in 56.7% of the CAD patients, and was associated with twice the odds of CAD compared to non-smokers. The effect estimate of smoking on CAD in our setting was of similar magnitude to reports from elsewhere [12, 13]. In a country like Jordan, where more than 40% of the adult population are smokers, the public health benefits of effective smoking cessation programs would be substantial. Indeed, in a study from USA, it was found that reduced smoking prevalence led to a 12% reduction in deaths from coronary disease from 1980 to 2000 [6]. Other benefits of smoking cessation, such as decreased prevalence of chronic obstructive pulmonary disease and lung cancer, would come in addition to this.

The other modifiable risk factor identified in our study was DM. Obesity has been steadily increasing in Middle Eastern countries, and has led to an epidemic of type 2 DM in the region. Indeed, the 13.7% prevalence of DM in the WHO Eastern Mediterranean Region is far above the global average of 8.5% [8]. In our study, individuals with DM had a 2.6 times increased odds of CAD, which is in line with studies from other parts of the world [14]. On the other side of the coin, improved glycaemic control and changes in lifestyle has been shown to effectively reduce long-term complications and improve prognosis in patients with impaired glucose tolerance or DM [15, 16]. Healthcare interventions to control the diabetes epidemic in the region, including early detection and management of individuals with impaired glucose tolerance, should be a top priority in the coming years. Furthermore, structural interventions to promote physical activity and a healthy diet, might have an even larger health effect on the population level. Indeed, a recent study from Mexico found that introduction of tax on sugar sweetened beverages led to a significant reduction in intake of taxed beverages followed by an increase in purchases of bottled plain water, showing that unhealthy habits can be modified by policy interventions [17].

Surprisingly, our study did not show any significant effect on CAD of previous hypertension, previous hyperlipidemia or a family history of CVD. It is possible that these conditions were underreported in our study; for example did only 5.7% of study participants report previous hyperlipidemia, which means that many might have been undiagnosed and misclassified. Moreover, our study might have been underpowered to detect more subtle increases in the odds of CAD. Hence, it is not possible to exclude the significance of these risk factors based on our study.

Our study had certain limitation. First, this was a hospital based study and the patients might not be representative of the general population. Second, as in any cross-sectional study, it is hard to make any firm conclusions about causality. However, the risk factors identified in our study were well in line with studies from elsewhere, and we believe the results are valid. Finally, there was a risk of misclassification bias, since information about risk factors was based on patient interviews. However, since misclassification bias is in direction of the null value [18], the positive findings from our study would rather be an underestimate of the true effect.

## Conclusions

In conclusion, age, gender, DM and smoking were strong and significant risk factors for CAD among Jordanian patients who underwent coronary angiography. Given the high prevalence of obesity, type 2 diabetes and smoking in the region, CAD is likely to continue to increase over the coming years. Public health interventions to reduce the prevalence of DM and smoking could have a huge public health impact in the region.

## Abbreviations

CAD: Coronary artery disease
CI: Confidence interval
CVD: Cardiovascular disease
DM: Diabetes mellitus
OR: Odds ratio
WHO: World health organization
